# MiR-23a transcriptional activated by Runx2 increases metastatic potential of mouse hepatoma cell via directly targeting Mgat3

**DOI:** 10.1038/s41598-018-25768-z

**Published:** 2018-05-09

**Authors:** Huang Huang, Yubo Liu, Peishan Yu, Jianhua Qu, Yanjie Guo, Wenli Li, Shujing Wang, Jianing Zhang

**Affiliations:** 1 School of Life Science and Medicine, Dalian University of Technology, Panjin, China; 2 Department of Biochemistry, Dalian Medical University, Dalian, China

## Abstract

MicroRNAs (miRNAs) and aberrant glycosylation both play important roles in tumor metastasis. In this study, the role of miR-23a in N-glycosylation and the metastasis of mouse hepatocellular carcinoma (HCC) cells was investigated. The miRNA expression array profiles that were confirmed by qPCR and Western blot analyses revealed higher miR-23a expression levels in Hca-P cells (with lymphatic metastasis potential) than in Hepa1–6 cells (with no lymphatic metastasis potential), while the expression of mannoside acetylglucosaminyltransferase 3 (Mgat3) was negatively associated with metastasis potential. Mgat3 is a key glycosyltransferase in the synthesis of the bisecting (β1,4GlcNAc branching) N-glycan structure. Bioinformatics analysis indicated that Mgat3 may be a target of miR-23a, and this hypothesis was verified by dual-luciferase reporter gene assays. Furthermore, we found that the transcription factor Runx2 can directly bind to the miR-23a gene promoter and promote its expression, as shown in dual-luciferase reporter gene assays and ChIP assays. Collectively, these results indicate that miR-23a might increase the metastatic potential of mouse HCC by affecting the branch formation of N-glycan chains presented on the cell surface through the targeting of the glycosyltransferase Mgat3. These findings may provide insight into the relationship between abnormal miRNA expression and aberrant glycosylation during tumor lymphatic metastasis.

## Introduction

The majority of cancer-related deaths are attributed to the metastatic spread of cancer cells to vital organs rather than to primary tumor outgrowth. Aberrant glycosylation, including the aberrant expression and glycosylation of mucins, on the cell surface is commonly observed during malignant transformation, as are abnormal branching of N-glycans and increased levels of sialic acid on proteins and glycolipids^1^. The structural variability of glycans is dictated by the tissue-specific regulation of glycosyltransferase genes, the availability of sugar nucleotides, and competition between enzymes for acceptor intermediates during glycan elongation^2^.

One widespread glycosylation change that promotes malignancy is the enhanced formation of β1,6-N-acetylglucosamine (β1,6GlcNAc) side chains caused by increased mannoside acetylglucosaminyltransferase 5 (Mgat5) activity and counteracting β1,4GlcNAc (the bisecting GlcNAc) branching of N-linked structures synthesized by Mgat3^3^. Mgat3 is a glycosyltransferase that catalyzes the transfer of GlcNAc in a β1,4 linkage to mannose on N-glycans, thus forming a bisecting GlcNAc structure, and Mgat3 has been regarded as a suppressor of metastasis with varying effects on cell adhesion and migration^4^.

MicroRNAs (miRNAs) are endogenous non-coding RNAs of approximately 21 nucleotides that have emerged as key post-transcriptional regulators of gene expression. Through binding to perfect or nearly perfect complementary sequences in the 3′ untranslated regions (UTRs) of target mRNAs, miRNAs can silence genes by either mRNA degradation or translational repression^5,6^. As a result, miRNAs are involved in multifarious cellular processes, including cell differentiation, proliferation and apoptosis, and function as either oncogenes or tumor suppressors in several human malignancies^7^. It is becoming increasingly evident that miRNAs play an important role in tumor metastasis. For example, miR-125a and miR-26a suppress tumor metastasis in hepatocellular carcinoma (HCC)^8,9^, while miR-203 suppresses cell proliferation, migration and invasion in colorectal cancer^10^. In our previous research, both let-7c and miR-34a were shown to inhibit the lymphatic metastasis potential of mouse HCC cells^11,12^. Furthermore, Brian E *et al*. reported that elevated serum miR-16, miR-378, and sICAM1 levels correlate with bone metastasis^13^. These findings highlight the potential of miRNA profiling in cancer therapeutic decision-making and in the diagnosis of tumor metastasis.

Given the important roles of miRNA and glycosylation of glycoproteins and glycolipids in malignancy, it is conceivable that miRNAs may play an important role in tumor progression by targeting specific glycosyltransferase that catalyze the formation of specific glycan structures. Some enzymes involved in protein glycosylation have been reported to be targets of cancer relevant miRNAs. miR-26a, miR-34a and miR-146a suppress HCC cell progression by targeting fucosyltransferase 8 (FUT8), the only enzyme responsible for β1,6-fucosylation of N-glycans^14^; O-GlcNAc transferase (OGT) was identified as a novel target of miRNA-7 in a mouse glioblastoma xenograft model^15^ and of miR-24-1 in human breast cancer cells^16^; mature miR-17-5p and passenger miR-17-3p induce HCC by targeting N-acetylgalactosaminyltransferase 7 (GALNT7)^17^; Let-7c inhibits metastatic ability of mouse hepatocarcinoma cells via targeting mannoside acetylglucosaminyltransferase 4 isoenzyme A (Mgat4a)^11^. However, the role of miRNAs in regulating glycosylation and tumor metastasis remains mostly unexplored.

Hca-P and Hepa1–6 are two mouse HCC cell lines. The Hca-P cell line, a lymphatic metastasizing clone isolated from the H22 cell line, forms lymphatic metastasis in 615-mice upon subcutaneous injection into the foot pad, while Hepa1–6 cells do not cause lymphatic metastasis^18^. We performed a miRNA microarray analysis to analyze the miRNA profiles in these two cell lines^12^, and found that miR-23a levels were significantly higher in Hca-P cells than in Hepa1–6 cells, which suggested the correlation between miR-23a expression and lymphatic metastasis in mouse HCC cells. Bioinformatics analysis predicted that miR-23a may regulate several glycosyltransferase-encoding genes, such as B3galt2, B3gnt1, Gxylt1, Gcnt4, B3gat2 and Mgat3^19^.

miR-23a has been implicated in several physiological and pathological processes, including osteoblast differentiation, cardiac hypertrophy, and muscular atrophy, and it has been reported as both an oncogene and tumor suppressor in tumorigenesis and development^20^. Gao *et al*. revealed that c-Myc transcriptionally represses miR-23a and miR-23b, which function as tumor suppressors, resulting in greater expression of their target protein, mitochondrial glutaminase, in human P-493 B lymphoma cells and PC3 prostate cancer cells^21^. In contrast, Berchem G *et al*. found that in hypoxic tumor-derived microvesicles, miR-23a operates as an immunosuppressive factor, because it directly targets CD107a expression in NK cells^22^. Coincidentally, Frampton *et al*. identified miR-23a promotes tumor progression via acting as cooperative repressors of a network of tumor suppressor genes in pancreatic ductal adenocarcinoma (PDAC) cells^23^.

miR-23a is the first member of the miR-23a~27a~24-2 cluster, which is well conserved among various species^20^, but the transcriptional regulation of this intergenic miRNA cluster is elusive. Lee *et al*. showed that like protein-coding genes, the transcription of the miR-23a~27a~24-2 cluster can be Pol II dependent and the promoter region covering −603~+36 bp lacks the common promoter elements, such as a TATA box, the initiator element, or the TFIIB recognition element^24^. Hernandez-Torres F *et al*. found that Srf is critical for miR-23a~27a~24-2 cluster expression, whereas other muscle-enriched transcription factors provide regulatory cues at both the transcriptional and post-transcriptional levels in cardiac and skeletal muscles^25^. Hassan M Q *et al*. showed that the miR-23a~27a~24-2 cluster is directly and negatively regulated by Runx2 in osteoblast differentiation^26^. However, these authors did not address its tissue-specific regulation, in particular with regard to HCC.

In the present study, we determined that affecting the branch formation of N-glycan chains by targeting Mgat3 might be one of the mechanisms by which miR-23a increases the metastatic ability of mouse HCC. In terms of the regulation of miR-23a expression, we showed that Runx2 enhances miR-23a expression via transcriptional activation of the miR-23a promoter in mouse HCC cells.

## Results

### miR-23a is upregulated in metastatic mouse HCC cell lines

**Figure 1:**
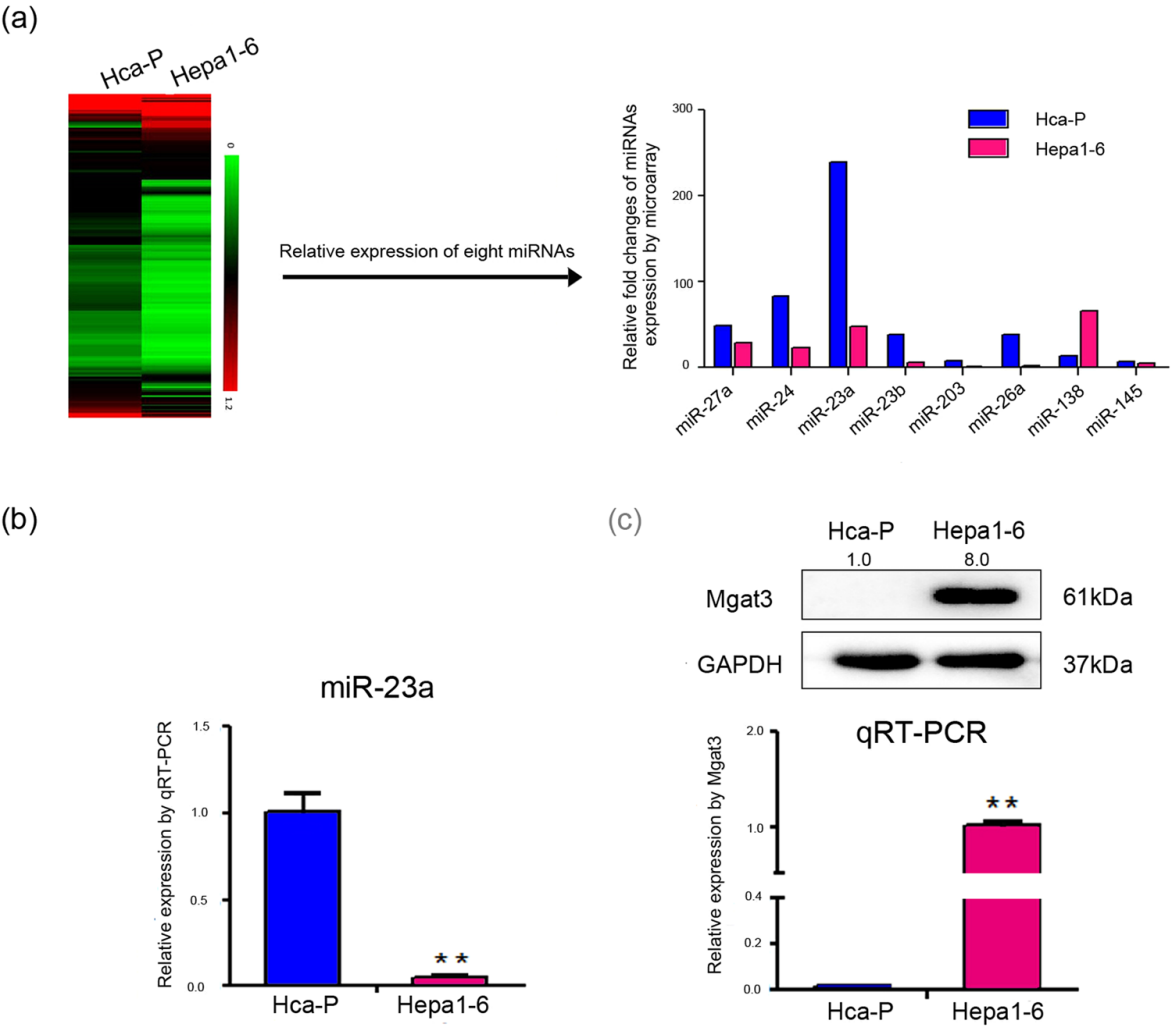
Constitutive expression of miR-23a and Mgat3 in mouse HCC cell lines. (**a**) miRNA microarray was performed to compare the miRNA expression profiles of Hca-P and Hepa1–6 cells (left)^27^. The relative expression of miR-23a and several miRNAs, which are related to tumor malignancy^9,10,16,20,21,28,29^, measured by microarray is displayed as a histogram (right). (**b**) The relative expression of miR-23a in Hca-P and Hepa1–6 cells as measured by qRT-PCR. (**c**) Mgat3 expression level in Hca-P and Hepa1–6 cells, as measured by Western blotting (upper) and qRT-PCR (below). The numbers upside WB figure present relative intensity of the bands normalized by corresponding GAPDH bands. Data are presented as the median with error bars (*p < 0.05; **p < 0.01).

Our miRNA microarray analyses showed that miR-23a expression levels were significantly higher in Hca-P cells (with lymphatic metastatic potential) than in Hepa1–6 cells (with no lymphatic metastatic potential)^27^, while the relative expressions of miR-23a were higher than several tumor malignancy related miRNAs^9,10,16,20,21,28,29^ in the two mouse HCC cell lines (Fig. 1a).

To probe the involvement of miR-23a in HCC metastasis, we measured miR-23a transcript levels by qPCR. The results were consistent with those of miRNA profiling (Fig. 1b), indicating the participation of miR-23a in tumor metastasis.

### Mgat3 is a direct target of miR-23a

**Figure 2:**
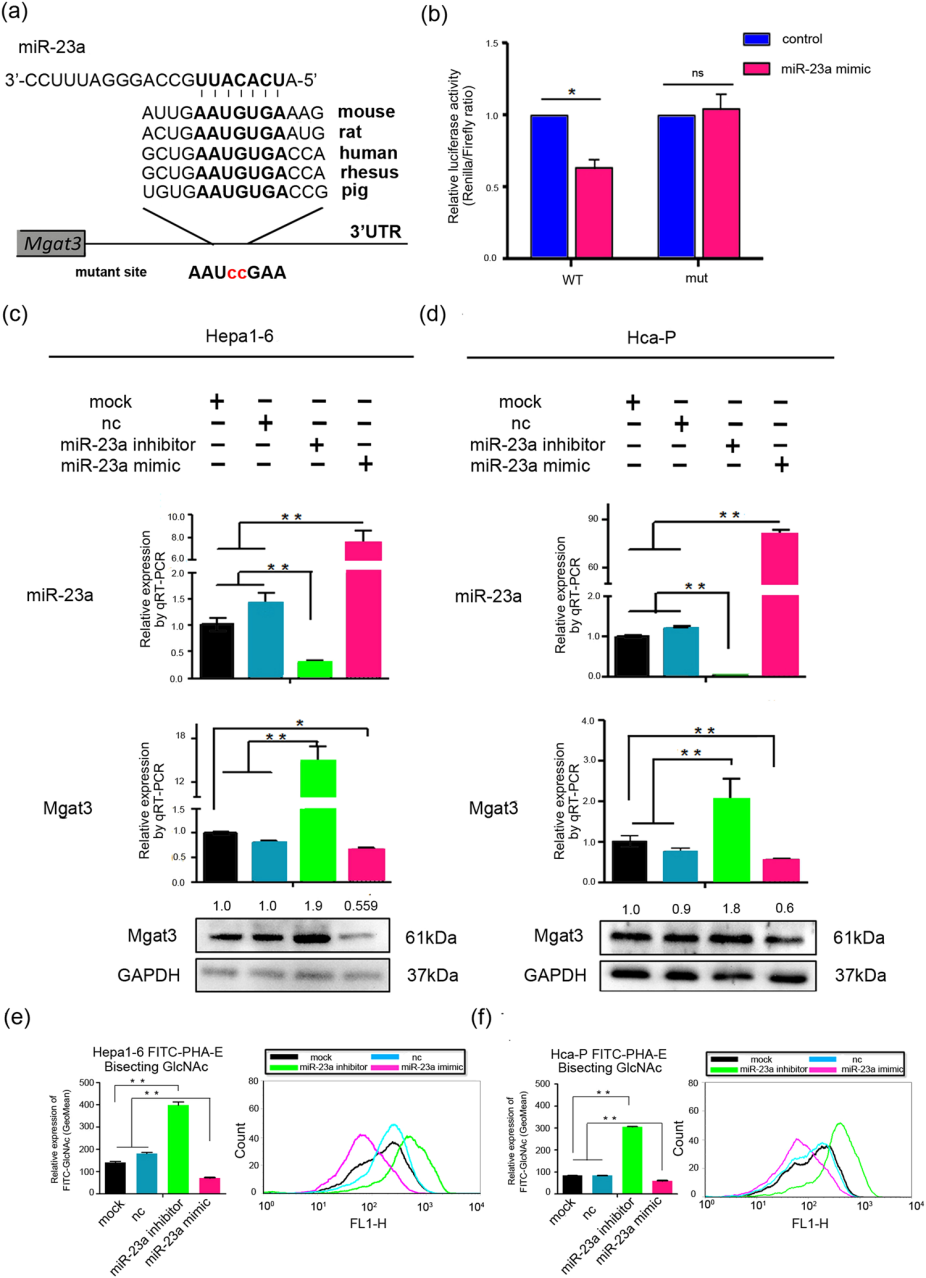
Mgat3 is a direct target of miR-23a. (**a**) The putative miR-23a-binding sequences in the 3′UTR of Mgat3 mRNA from several species are shown^19^. A mutation was generated in the Mgat3 3′UTR sequence at the complementary site for the seed region of miR-23a (red). (**b**) The luciferase reporter assays show reporter activity after co-transfection of either psiCHECK-2-Mgat3-3′UTR (2180–2751 bp) or psiCHECK-2-mutant Mgat3 3′UTR with miR-23a mimic in Hepa1–6 cells. (**c**,**d**) miR-23a and Mgat3 expression levels in mouse HCC cells transfected with miR-23a mimic or miR-23a inhibitor relative to those transfected with CP transfection reagent only (mock) or scrambled miR-23a (nc) as measured by qRT-PCR and Western blotting. The numbers upside WB figure present relative intensity of the bands normalized by corresponding GAPDH bands. Data are presented as the median with error bars (*p < 0.05; **p < 0.01). (**e**,**f**) FCM analysis of the levels of bisecting structures recognized by FITC-PHA-E on the cell surface of Hepa1–6 and Hca-P cells transfected with CP transfection reagent only (mock), scrambled miRNA (NC), miR-23a mimic or miR-23a inhibitor. See also Supplementary Fig. S2 for additional FCM analysis of the levels of β-1,6 branching of N-glycans.

To ascertain the potential mechanism by which miR-23a affects tumor metastasis, we searched for potential target genes of miR-23a using two publicly available databases, TargetScan^19^ and miRanda^30^. Mgat3 was identified as a potential candidate; it is an important suppressor of metastasis in several types of tumors, and its mRNA and protein expression levels were negatively correlated with miR-23a expression in Hca-P and Hepa1–6 cells (Fig. 1c). To identify the potential binding site, we inserted a wild-type or mutant 3′UTR sequence (571 bp, at 2180~2751 bp) immediately downstream of the luciferase reporter gene (Fig. 2a) and co-expressed the resulting plasmids with either miR-23a mimic or scrambled miRNA in Hepa1–6 cells. As shown in Fig. 2b, miR-23a significantly suppressed relative Renilla luciferase activity compared with scrambled miRNA, whereas luciferase activity did not decrease in the presence of the mutant 3′UTR reporter, indicating that functionality depends on an intact seed sequence. The specificity of miR-23a-Mgat3 3′UTR binding was also verified by the results of the Mgat4a and Mgat5 3′UTR control groups (see Supplementary Fig. S1).

Moreover, miR-23a overexpression significantly decreased Mgat3 expression at both the mRNA and protein levels compared with the controls in both mouse HCC cell lines. In contrast, miR-23a downregulation with the miR-23a inhibitor significantly increased Mgat3 expression (Fig. 2c,d).

Overall, these results strongly support the direct suppression of Mgat3 by miR-23a, which is likely to contribute to promoting metastasis.

Aberrant glycosylation affects tumor invasion and metastasis and is regarded as a hallmark in certain types of tumors. Mgat3 can specifically catalyze bisecting structures in N-glycans displayed on the cell membrane, while β-1,6 branching of N-glycans can be catalyzed by Mgat5. To further determine the role of Mgat3 in the miR-23a-mediated promotion of metastasis, we analyzed N-linked glycosylation on the cell membrane of miR-23a-transfected mouse HCC cells using flow cytometry (FCM) analysis by labeling specific N-glycans with fluorescein isothiocyanate lectins (FITC-PHA-E and FITC-PHA-L). miR-23a overexpression suppressed the levels of bisecting structures in N-glycans, and conversely, miR-23a downregulation increased the levels of these bisecting structures (Fig. 2e,f). In addition, there was no significant difference in the levels of β-1,6 branching of N-glycans (see Supplementary Fig. S2). The results were consistent with the expression levels of Mgat3.

### miR-23a modulates mouse HCC cell migration and invasion *in vitro* and *in vivo*

**Figure 3:**
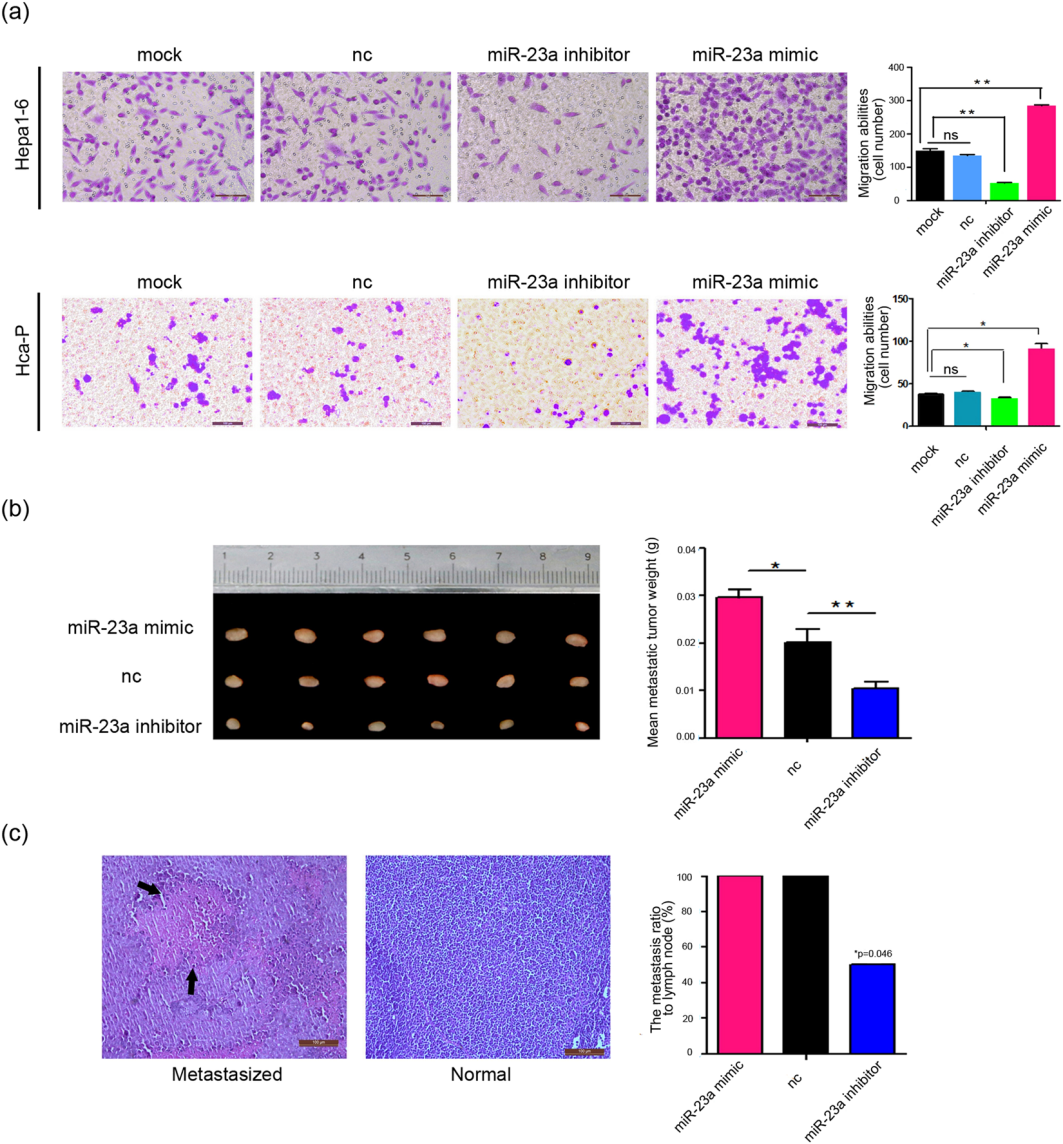
miR-23a promotes cell migration and invasion. (**a**) Transwell migration assay with mouse HCC cells transfected with CP transfection reagent only (mock), scrambled miRNA (NC), miR-23a mimic or miR-23a inhibitor. Representative pictures of migrated cells (right) and quantification of the number of tumor cells (left). The fields of view were randomly selected under a microscope, and the micrograph scale bars represent 100 μm. Similar transwell invasion assay results were obtained with Hca-P cells (see Supplementary Fig. S3). (**b**) Three groups of 615-mice were injected subcutaneously with Hca-P/miR-23a mimic, Hca-P/miR-scramble (nc), or Hca-P/miR-23a inhibitor cells. After 4 weeks, the mice were sacrificed, and the inguinal lymph nodes were isolated and weighted. The Hca-P/miR-23a mimic group showed a significant increase in mean lymph node weight compared with the control group, while the Hca-P/miR-23a inhibitor group showed a decrease. (**c**) The inguinal lymph nodes were sectioned and stained with hematoxylin and eosin. Representative pictures of HE staining showed metastatic lesions (black arrow) and normal tissue in the lymph node sections. The lymph node metastasis rate was significantly lower in the Hca-P/miR-23a inhibitor group than in the other groups (chi-square test; *p = 0.0455; p < 0.05), as shown in the histogram. The micrograph scale bar represents 100 μm.

Next, we explored the effect of miR-23a on cell migration and invasion *in vitro* using transwell chambers with or without Matrigel. Transwell assays without Matrigel clearly indicated that miR-23a mimic transfection promoted the migration of Hca-P and Hepa1–6 cells compared with control transfections (Fig. 3a). In addition, the invasiveness of miR-23a mimic-transfected Hca-P cells was enhanced, as demonstrated by transwell assays with Matrigel. In contrast, transfection with the miR-23a inhibitor had the opposite effects (see Supplementary Fig. S3).

Then, the effect of miR-23a on the lymph node metastasis of Hca-P cells in 615-mice was examined. The mean weight of the inguinal lymph nodes (location of potential metastasis) was significantly increased in the miR-23a mimic-transfected group but was lighter in the miR-23a inhibitor-transfected group than in the control group (Fig. 3b). Observation of lymph node HE-stained sections revealed aberrant swollen oval-like morphology, follicular diffuse fusion or diffuse invasion of lymphoma cells in the three groups, while the lymph node metastasis rate was significantly lower in the Hca-P/miR-23a inhibitor group than in the other groups (3/6 compared to 6/6). Representative images are shown in Fig. 3c. The *in vivo* results suggest that increased miR-23a levels can promote lymph node metastasis, while decreased miR-23a levels protect the lymph node invasion.

Together, these results indicate that miR-23a significantly enhances mouse HCC cell migration and invasion *in vitro* and *in vivo*.

### miR-23a is positively and direct regulated by Runx2

**Figure 4:**
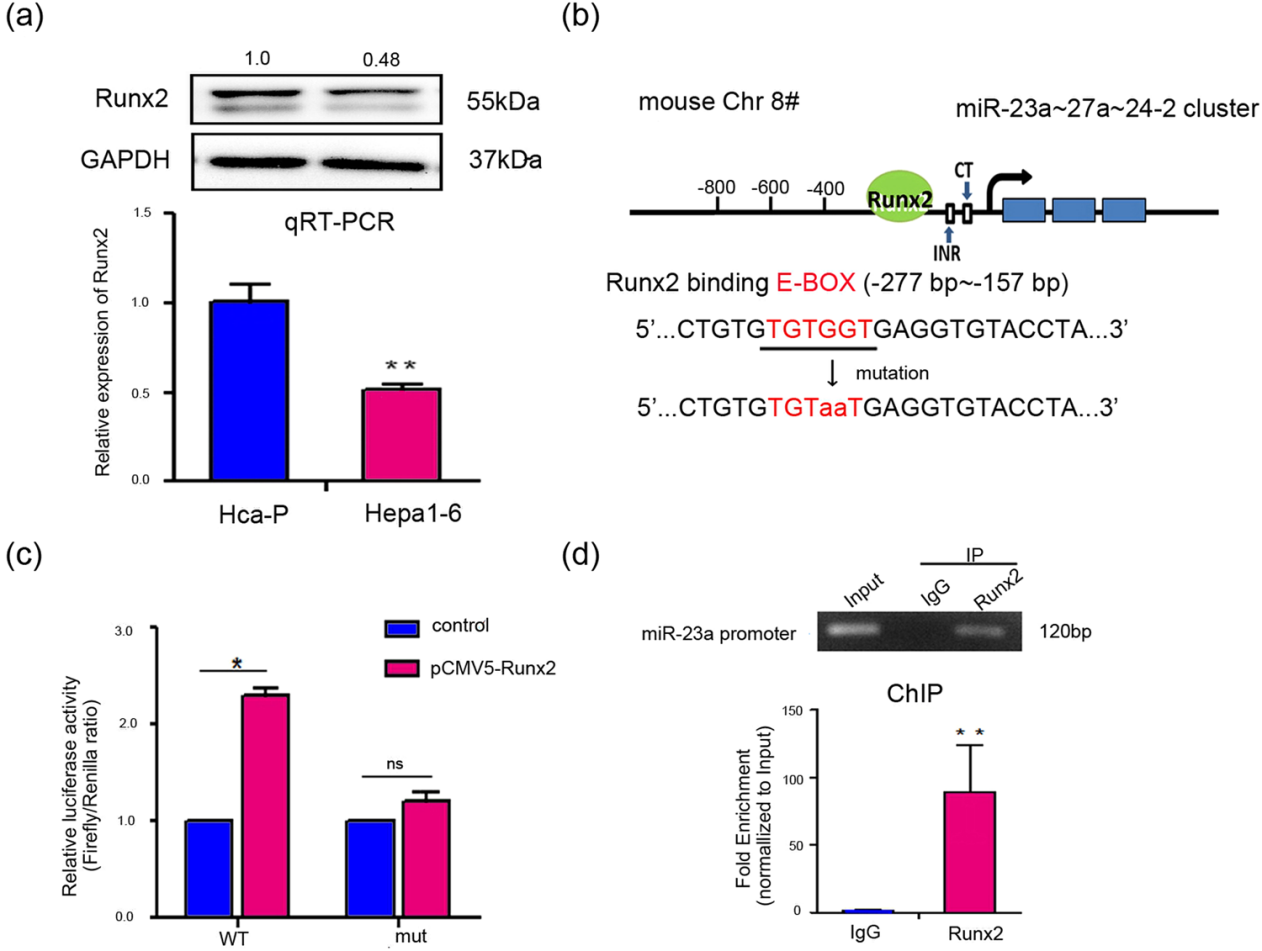
miR-23a is positively and direct regulated by Runx2. (**a**) Runx2 expression level in Hca-P and Hepa1–6 cells, as measured by Western blotting (upper) and qRT-PCR (lower). The numbers upside WB figure present relative intensity of the bands normalized by corresponding GAPDH bands. (**b**) The putative Runx2-binding sequence (E-BOX) in the miR-23a gene promoter is shown. A mutation was generated in the miR-23a promoter at the complementary site for the E-BOX of Runx2 (red). (**c**) Luciferase assays of reporter activity after co-transfection of either the partial miR-23a promoter or the mutant promoter with the pCMV-Runx2 plasmid into Hepa1–6 cells. (**d**) ChIP assays were performed according to the EZ-Magna ChIP^TM^ protocol using chromatin from Hepa1–6 cells, and both anti-Runx2 antibody and normal rabbit IgG were used as the immunoprecipitating antibodies in an overnight incubation. Purified DNA was then analyzed by qRT-PCR (upper) and RT-PCR (lower) using primers specific for the miR-23a promoter. For the ChIP assay positive controls, see also Supplementary Fig. S4.

In continuing to explore the regulatory mechanism of miR-23a biosynthesis, we searched for potential transcription factors that may bind the −0.801-kb fragment of the mouse miR-23a promoter by using two publicly available databases, TRANSFAC^31^ and TESS^32^. “TGTGGT”, located at −203 to −198 bp in the miR-23a promoter, was predicted as the binding site for Runx2 (Fig. 4b). Runx2 mRNA and protein expression levels were positively correlated with miR-23a expression levels in Hca-P and Hepa1–6 cells (Figs 1b and 4a).

To identify the potential binding site, we replaced the SV40 promoter (upstream of the firefly luciferase reporter gene in the pGL3-control vector) with wild-type or mutant promoter sequences (−801 bp to 0) and co-expressed these plasmids with the pCMV-Runx2 expression plasmid in Hepa1–6 cells. As shown in Fig. 4c, Runx2 significantly increased relative firefly luciferase activity compared with the control, whereas the mutant promoter reporter had no such effect, indicating that Runx2 upregulates miR-23a transcription by directly binding to the miR-23a promoter.

ChIP assays were performed to verify the interaction of Runx2 with the miR-23a promoter in Hepa1–6 cells. The associated DNA fragments were detected by RT-PCR and qPCR (Fig. 4d), and the results showed that Runx2 was able to bind to the −277 bp to −157 bp region upstream of the miR-23a gene. The interaction between Histone H3 and the Gapdh promoter was also detected as positive control in ChIP assay (see Supplementary Fig. S4).

**Figure 5:**
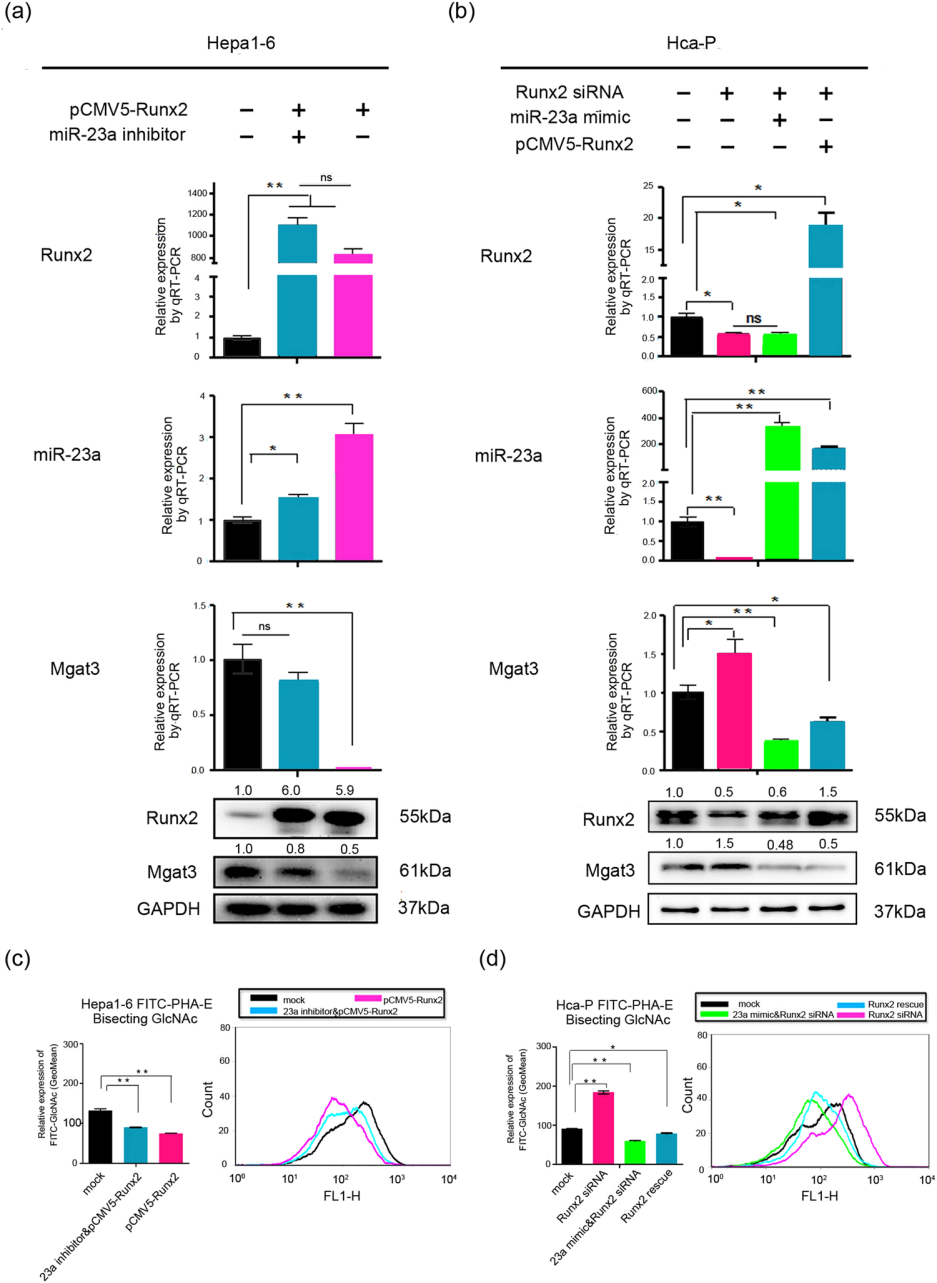
Runx2 regulates Mgat3 expression via miR-23a. (**a**) Runx2, miR-23a and Mgat3 expression levels were measured by qRT-PCR (upper) and Western blot (lower) after transfection of 3.6 µg of pCMV-Runx2 plasmid or 150 nM miR-23a inhibitor and 3 µg of pCMV-Runx2 plasmid into Hepa1–6 cells relative to transfection of CP transfection reagent only (mock). (**b**) Runx2, miR-23a and Mgat3 mRNA levels were determined in Hca-P cells transfected with 100 nM Runx2 siRNA, 100 nM Runx2 siRNA and 3 µg of pCMV-Runx2 plasmid (Runx2 rescued after 24 h), or 150 nM miR-23a mimic and 100 nM Runx2 siRNA relative to the mock control. The numbers upside WB figure present relative intensity of the bands normalized by corresponding GAPDH bands. (**c**) FCM analysis of the levels of bisecting structures on the cell surface recognized by FITC-PHA-E after transfection of Hca-P cells with 100 nM Runx2 siRNA, 100 nM Runx2 siRNA and 3 µg of pCMV-Runx2 plasmid (Runx2 rescued after 24 h), or 150 nM miR-23a mimic and 100 nM Runx2 siRNA relative to the mock control. (**d**) FCM analysis of the levels of bisecting structures on the cell surface recognized by FITC-PHA-E after transfection of Hepa1–6 cells with 3.6 µg of pCMV-Runx2 plasmid or 150 nM miR-23a inhibitor and 3 µg pCMV-Runx2 plasmid relative to the mock control. See also Supplementary Fig. S2 for additional FCM analysis of the levels of β-1,6 branching of N-glycans. Data are presented as median with error bars (*p < 0.05; **p < 0.01; ns p > 0.05).

Next, we detected the transcriptional activity of Runx2. As shown in Fig. 5a, overexpression of Runx2 increased miR-23a expression, while suppression of Runx2 decreased miR-23a levels. Moreover, Runx2 rescue 24 h after Runx2 siRNA transfection restored miR-23a expression, suggesting that Runx2 positively regulates miR-23a as a transcriptional activator (Fig. 5b).

Furthermore, we evaluated Mgat3 expression (Fig. 5a,b) and N-glycan structures (Fig. 5c,d) on the cell surface after Runx2 or miR-23a knockdown or overexpression. The Runx2 overexpression-mediated downregulation of Mgat3 expression and bisecting structures in N-glycans was weakened in the presence of the miR-23a inhibitor, while transfection of the miR-23a mimic diminished the Runx2 siRNA-mediated increase in the levels of Mgat3 expression and bisecting structures. In addition, there was no significant difference in the levels of β-1,6 branching of N-glycans (see Supplementary Fig. S2).

Taken together, these results show that Runx2 might suppress Mgat3 expression and bisecting structures in N-glycans by transcriptionally activating miR-23a.

## Discussion

It is well known that miRNAs can function as potential oncogenes or tumor suppressors by modulating the expression patterns of essential genes involved in tumorigenesis and metastasis^33^.

Aberrant glycosylation accompanies malignant transformation in all types of human cancer^34^. Given the important roles of miRNA and glycosylation of glycoproteins and glycolipids in malignancy, it is conceivable that miRNAs may play an important role in tumor progression by targeting specific glycosyltransferases that catalyze the formation of specific glycan structures.

Hca-P and Hepa1–6 are two mouse HCC cell lines. The Hca-P cell line, a lymphatic metastasizing clone isolated from the H22 cell line, forms lymphatic metastasis in 615-mice upon subcutaneous injection into the foot pad, while Hepa1–6 cells do not cause lymphatic metastasis^18^. We performed a miRNA microarray analysis to analyze the miRNA profiles in these two cell lines^12^ and found that miR-23a levels were significantly higher in Hca-P cells than in Hepa1–6 cells, which were identified by qRT-PCR (Fig. 1b). Bioinformatics analysis revealed that miR-23a may regulate several glycosyltransferase-encoding genes, such as B3galt2, B3gnt1, Gxylt1, Gcnt4, B3gat2 and Mgat3^19,30^. Based on our previous finding that let-7c inhibits the metastatic ability of mouse HCC cells via targeting Mgat4a^11^, we focused in this study on Mgat3, which was negatively correlated with miR-23a expression in mouse HCC cells (Fig. 1b,c) and has been regarded as a metastases suppressor, as the predicted target of miR-23a. To confirm that Mgat3 is a target of miR-23a, we showed the direct binding of miR-23a to the Mgat3 3′-UTR in luciferase reporter assays (Fig. 2a,b) and verified miR-23a could suppress Mgat3 expression both in transcriptional and translational level by transfection experiments (Fig. 2c,d). Further studies demonstrated that miR-23a overexpression decreased bisecting N-glycan structures on the cell surface (Fig. 2e,f) and promoted cell migration and invasion *in vitro* and lymphatic metastasis *in vivo* (Fig. 3). All these results indicate that miR-23a may promote metastatic activity by, at least in part, repressing Mgat3 activity in the N-glycan pathway in mouse HCC cells.

miR-23a has been implicated in several physiological and pathological processes, including osteoblast differentiation, cardiac hypertrophy, and muscular atrophy, and it has been reported as both an oncogene and tumor suppressor gene in tumorigenesis and development^23^. The controversial role of miR-23a in different kinds of cancer might be a result of differential regulation of miR-23a expression in a tissue- and time-dependent manner.

miR-23a is the first member of the miR-23a~27a~24-2 cluster, which is well conserved among various species^20^. The transcriptional regulation of the intergenic miR-23a~27a~24-2 cluster is elusive, although its promoter region (from −603 to +36 bp) has been uncovered, it lacks the common promoter elements, such as TATA box or the TFIIB recognition element^24^, only Srf and Runx2 were found to be its transcriptional factors respectively in cardiac muscles and osteoblast^25,26^. In this study, we focused on the tissue-specific transcriptional regulation of miR-23a, in particular with regard to HCC.

After predicting transcription factors that may bind to the miR-23a promoter region by online bioinformatics analysis^31,32^, we focused on Runx2, which showed positive correlation with metastasis^35,36^ in HCC and found a positive correlation between Runx2 expression and miR-23a~27a~24-2 cluster levels in these two mouse HCC cell lines (Figs 1b and 4a). The direct upregulation of the miR-23a~27a~24-2 cluster by Runx2 in mouse HCC cells was verified by luciferase reporter assays and ChIP (Fig. 4b–d). Furthermore, transfection experiments were carried out to verify that Runx2 suppressed the Mgat3 expression as well as the bisecting structures in N-glycans by promoting the transcriptional activity of miR-23a (Fig. 5). Considering the oncogenic role of miR-23a, the promotion of miR-23a expression by Runx2 plays a carcinogenic role in mouse HCC cells, as it does in several cancer types, including ovarian, breast, liver and prostate cancer^37–40^.

**Figure 6:**
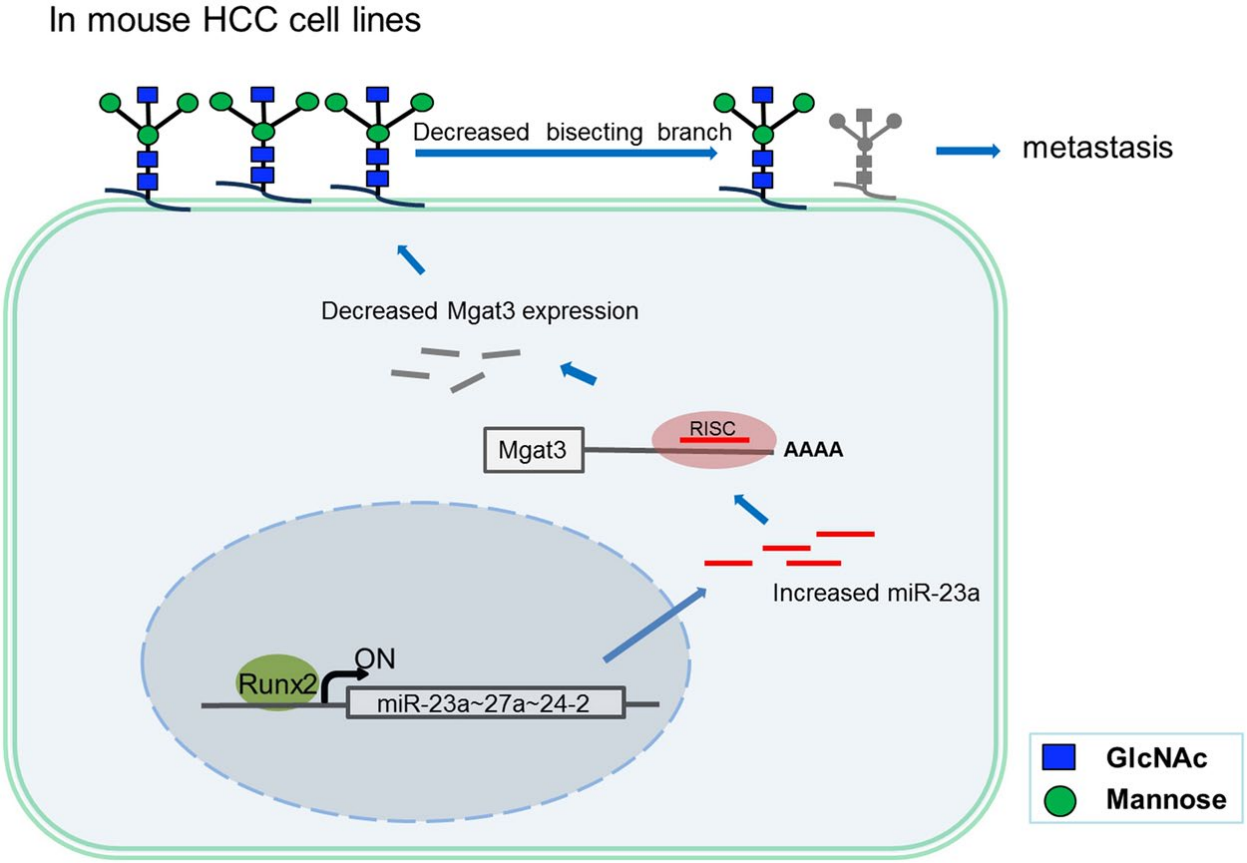
Model for the mechanism by which miR-23a promotes tumor metastasis by changing N-glycan branching on the cell surface. In mouse HCC cells, high expression levels of the transcription factor Runx2 activate the transcription of the miR-23a∼27a∼24-2 cluster by binding to its promoter (around −277 bp to −157 bp). Then, increased miR-23a levels restrain the expression of the glycosyltransferase Mgat3, which catalyzes the branch formation of bisecting β1,4-GlcNAc. Finally, the decrease in bisecting structures of N-glycans on the cell surface plays a positive role in metastasis.

In summary, our findings identify Runx2 as a transcriptional activator of miR-23a and demonstrate that as an oncogene, miR-23a may promote lymphatic metastasis by targeting Mgat3, which regulates the branching pattern of N-glycans on the mouse hepatoma cell surface (Fig. 6).

This work provides insight into the molecular mechanisms by which miR-23a promotes metastasis and reveals an inverse correlation between miR-23a expression and bisecting N-glycan structure levels. Targeting the interaction between miR-23a and Mgat3 would be a potential therapeutic approach to blocking cancer cell metastasis.

## Methods

### Cell culture and animals

The Hepa1–6 and Hca-P cell lines were used in this study. Hepa1–6 cells are a standard HCC cell line that does not cause lymphatic metastasis in inbred 615-mice upon subcutaneous injection into the foot pad, while the Hca-P cell line was isolated from the ascites-type HCC cell line H22 as a lymphatic metastasizing clone. The two HCC cell lines are suitable for our study of the mechanism of tumor lymph node metastasis.

The non-metastatic mouse HCC Hepa1–6 cell line was obtained from the Cell Bank of Peking Union Medical University (Beijing, China) and cultured in 90% DMEM (Gibco) supplemented with 1% penicillin/streptomycin (Beyotime, China) and 10% fetal bovine serum (Gibco, USA). The mouse HCC Hca-P cell line, which has metastatic potential in the lymph nodes, (established and stored by Department of Pathology, Dalian Medical University) was subcutaneously injected into abdominal cavity of 615-mice and grown in abdominal cavity about 7 days, then mice were sacrificed and Hca-P cells enriched from ascites were cultured in 90% RPMI-1640 (Gibco, USA) supplemented with 1% penicillin/streptomycin antibiotics (Beyotime, China) and 10% fetal bovine serum (Gibco, USA). All cells were cultured in a humidified incubator (Thermo, USA) at 37 °C with 5% CO_2_. Inbred 615-mice (5-week old males) were purchased from the Animal Facility of Dalian Medical University.

The authors confirm that all methods were carried out in accordance with written consent under approval of the Dalian University of Technology Review Board, and all experimental protocols were approved by the Medical Ethics Committee of School of Life Science & Medicine.

### Quantitative RT-PCR

Total RNA was extracted using TRIzol reagent (Invitrogen, USA). First-strand cDNA corresponding to mature miR-23a and U6 was synthesized using a PrimeScript^TM^ RT reagent kit (TaKaRa, Japan) and specific stem loop primers. First, the mixture containing total RNA and specific primers was heated to 70 °C for 10 min to melt RNA secondary structure. After adding SuperScript II and RNasin, the mixtures were incubated for 60 min at 42 °C and for 10 min at 72 °C in a 20-μl reaction volume. Prepared cDNA was analyzed in triplicate 20-μl reactions (20 μmol/L primers and 10 μl of Master Mix) by adapting the standard protocol provided with the SYBR Green PCR Master Mix (TaKaRa, Japan). The default PCR procedure was used on a Roche LightCycler 96. The relative expression levels of each gene were calculated and normalized relative to U6 or Gapdh expression levels using the 2^−ΔΔCt^ method. The RT-PCR primers were listed below:

Gapdh sense: 5′-accacagtccatgccatcac-3′, antisense: 5′-tccaccaccctgttgctgta-3′; Runx2 sense: 5′-cctcagtgatttagggcgca-3′, antisense: 5′-gtggtggagtggatggatgg-3′; Mgat3: sense 5′-agtgggttgagtgtgtgtgc-3′, antisense: 5′-ctcgtggttgatgttgatgg-3′; Mgat5 sense: 5′-ggtgtcctcgtttaccttgg-3′, antisense: 5′-ctcctcgtgcttcttcatca-3′; MiR-23a sense: 5′-atcacattgccagggatt-3′, antisense: 5′-ctcaactggtgtcgtgga-3′. RT-specific forward and reverse primers for qPCR analysis of miR-23a were designed as part of the Bulge-Loop™ miRNA qRT-PCR primer set (Ribo-bio, China).

### Western blotting (WB)

Cells were lysed with RIPA lysis buffer (Beyotime, China) containing a protease inhibitor (Roche, Switzerland). Equal amounts of protein were separated by 10% SDS-PAGE and transferred to polyvinylidene difluoride membranes (Millipore, USA). The membranes were blocked with 5% nonfat milk in TBS for 2 h at room temperature and then probed with antibodies against Mgat3 (Abcam, USA), GAPDH (CST, USA), and Runx2 (CST, USA). After being washed, the membranes were incubated with horseradish peroxidase-conjugated secondary antibodies. Chemiluminescent detection was performed using an ECL kit (GE Healthcare, USA). Gapdh was detected on the same membrane as a loading control. Original WB pictures were shown in Supplementary Figs 5–10.

### Luciferase reporter assay

To test the direct binding of miR-23a to the 3′UTR of Mgat3 mRNA, one partial 3′-UTR sequence (571 bp, at 2180~2751 bp) of mouse Mgat3 containing the predicted miR-23a binding sites was amplified from normal mouse cDNA (sense primer 5′-ctcgagcagggctcctgcccacaagt-3′ and antisense primer 5′-gcggccgcagggtgaaatgaaattcaacc-3′) and cloned into the *XhoI/NotI* sites downstream of the Renilla luciferase stop codon in the psiCHECK^TM^-2 vector (Promega, USA). Partial sequences of the Mgat4a 3′-UTR (3–648 bp) and Mgat5 3′-UTR (1–1178 bp) were also cloned into the psiCHECK^TM^-2 vector as controls. The correct clones were confirmed by sequencing analysis. The corresponding mutant construct was created by mutating the seed region from 5′-AATGTGAA-3′ to 5′-AATccGAA-3′. To test the interaction between the 3′-UTR of Mgat3 and miR-23a in Hepa1–6 cells, 100 nM miR-23a mimic or scrambled miRNA was co-transfected with 100 ng of wild-type 3′UTR or mut 3′UTR. After 24 h, Hepa1–6 cell lysates from all treated groups were collected by using Passive Lysis Buffer (Promega, USA). Renilla luciferase activity was analyzed relative to firefly luciferase activity in the same sample by using the psiCHECK^TM^-2 vector (Promega, USA).

To test the direct binding between Runx2 and the miR-23a gene promoter, one section of the miR-23a promoter (−801 bp to 0) was amplified from normal mouse cDNA (sense primer 5′-gggaagcttgagccaccaactgca-3′ and antisense primer 5′-gggggatccaggcacagtgagggg-3′) and cloned into the *HindIII/BamHI* sites to replace the SV40 promoter upstream of the firefly luciferase reporter gene in the pGL3-control vector (Promega, USA). The correct clones were confirmed by sequencing analysis. The corresponding mutant construct was created by mutating the Runx2 E-BOX from 5′-TGTGGT-3′ to 5′-TGTaaT-3′. Firefly luciferase activity was analyzed relative to Renilla luciferase activity in the same sample by using the Dual-Luciferase Reporter Assay System (Promega, USA).

### Transient transfection

Hepa1–6 and Hca-P cells were cultured in six-well plates for 12–24 h before transfection. miR-23a mimic, miR-23a inhibitor (antisense oligonucleotide) and scrambled miRNA oligonucleotide pairs were purchased from Ribo-bio (Guangzhou, China). Runx2 siRNAs were designed and synthesized by GenePharma (Shanghai, China). Both cell lines were transfected with 100 nM miR-23a mimic, miR-23a inhibitor, scrambled miRNA or Runx2 siRNA using riboFECT™ CP transfection reagent (Ribo-bio, China) according to the manufacturer’s instructions. The eukaryotic expression plasmid pCMV-Runx2 was obtained from the Karsenty Lab (Columbia University) and was transfected into Hepa1–6 and Hca-P cells using Lipofectamine 2000 (Invitrogen, USA) according to the standard protocol. Cells were collected 48 h after transfection for further assays.

### *In vitro* migration and invasion assays

In the transwell assay, 5 × 10^4^ cells in 100 µl of serum-free medium were added to the upper chamber of an insert (8-μm pore size, Corning) coated with or without Matrigel (BD), while the lower surface contained complete culture medium supplemented with 10% FBS. Given the stimulatory effect of miR-23a on cell proliferation, cells were pre-treated with 5 μM mitomycin-C for 1 h before being seeded. After 48 h, the cells on the upper surface were removed, whereas the cells on the lower surface were fixed and stained with 0.05% crystal violet for 1 h. Finally, the cells that had passed through the upper chamber were counted under a DMI4000 B microscope (Leica, Germany), and the relative cell number was calculated.

### *In vivo* tumor metastasis assay

Eighteen inbred 615-mice were equally divided into three groups. Hca-P cells (5 × 10^6^) that had been transiently transfected with the miR-23a mimic, miR-23a inhibitor or scrambled miRNA were inoculated subcutaneously into the left footpad of each mouse. After 4 weeks, the mice were sacrificed, and their left inguinal lymph nodes were isolated, weighed, sectioned and stained with hematoxylin and eosin (HE). Sectioning and staining were completed with assistance from the Pathology Lab of Dalian Medical University.

### Chromatin immunoprecipitation assays

ChIP assays were performed in Hepa1–6 cells using EZ-Magna ChIP^TM^ A (Cat. #17–408, Millipore) following the standard protocol. The protein-DNA complexes were incubated with 5 µg of anti-Runx2 antibody (CST, #8486) or 5 µg of normal rabbit IgG (provided in the EZ-Magna ChIP^TM^ A kit) as the immunoprecipitating antibody. Purified DNA was subjected to RT-PCR (ExTaq, Takara) and qPCR (SYBR Green PCR Master Mix, TaKaRa) analyses using Runx2 ChIP primers. The positive control was incubated with 5 µg anti-acetyl Histone H3 (provided by EZ-Magna ChIP^TM^ A) and purified DNA was detected by RT-PCR using Gapdh ChIP primers.

The following primers were used: Runx2 ChIP sense primer 5′cttggaagtagaggagggctagg-3′; Runx2 ChIP antisense primer 5′-cctctacctctggagtctaggat-3′; Gapdh ChIP sense primer 5′-agagagggaggaggggaaat-3′; and Gapdh ChIP antisense primer 5′-gccctgcttatccagtccta-3′. Un-cut DNA electrophoretogram (RT-PCR) was shown in Supplementary Fig. S11.

### Flow cytometry (FCM)

Mouse HCC cells (1 × 10^6^) were incubated for 0.5 h at 4 °C in the dark with FITC-PHA-E or FITC-PHA-L (Vector Laboratories), which recognizes the β-1,4GlcNAc or β-1,6GlcNAc branching structure of N-glycans, respectively, at a final concentration of 20 µg/ml. Before FCM analysis, cells were washed twice with PBS and fixed with 50 µl of 1% paraformaldehyde.

### Statistical analysis

Each assay was performed at least three times. Data are presented as the mean ± S.D. Student’s t-test was used to determine the significance of data. P values < 0.05 indicated statistical significance. All the statistical analyses were performed with GraphPad Prism software.

## Electronic supplementary material

Supplementary Information
