# Supplementary material for: MiR-23a transcriptional activated by Runx2 increases metastatic potential of mouse hepatoma cell via directly targeting Mgat3
Source: Sci Rep. 2018 May 9;8:7366. doi: 10.1038/s41598-018-25768-z (PMC5943354; doi:10.1038/s41598-018-25768-z)
Supplement: Supplementary file 1 — Supplementary Information [file 41598_2018_25768_MOESM1_ESM.pdf]

Supplementary Information

MiR-23a transcriptional activated by Runx2 increases metastatic potential of mouse hepatoma cell via directly targeting Mgat3

Huang Huang[1], Yubo Liu[1], Peishan Yu[1], Jianhua Qu[2], Yanjie Guo[2], Wenli Li[1], Shujing Wang[2], Jianing Zhang[1, *]

[1] School of Life Science and Medicine, Dalian University of Technology, Panjin, China.

[2] Department of Biochemistry, Dalian Medical University, Dalian, China.

*Corresponding author: Jianing Zhang, School of Life Science and Medicine, Dalian University of Technology, Panjin, 122406, China; Tel.: +86 427 2631889; Fax: +86 427 2631889; E-mail address: jnzhang@dlut.edu.cn

Supplementary Results

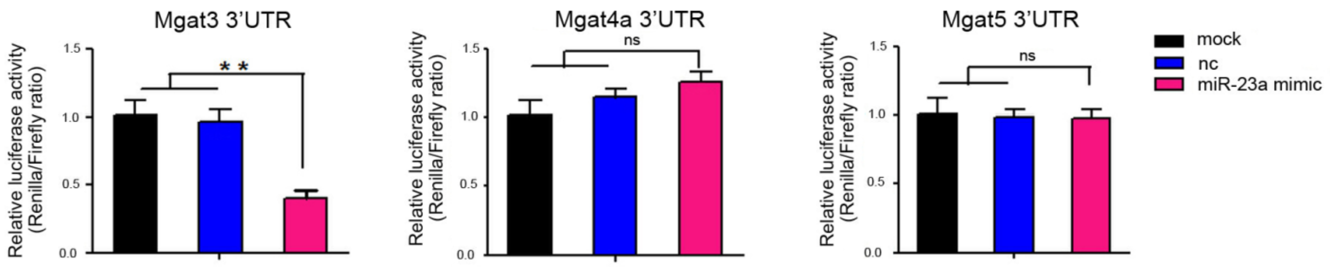

Supplementary Figure 1: miR-23a inhibits Mgat3 expression by specifically binding to its 3'UTR The luciferase reporter assays show the reporter activity (Renilla/firefly luciferase activity ratio) after co-transfection of Hepa1-6 cells with psiCHECK-2-Mgat3-3′UTR (2301-2605 bp), psiCHECK-2-Mgat4a-3′UTR (3-648 bp) or psiCHECK-2-Mgat5-3′UTR (1-1178 bp) and the miR-23a mimic. The reporter activity of the miR-23a mimic group is presented relative to that of the CP transfection agent only control group (mock) and the scrambled miR-23a group (nc). Data are presented as the median with error bars (**$p < 0.01$; ns $p > 0.05$).

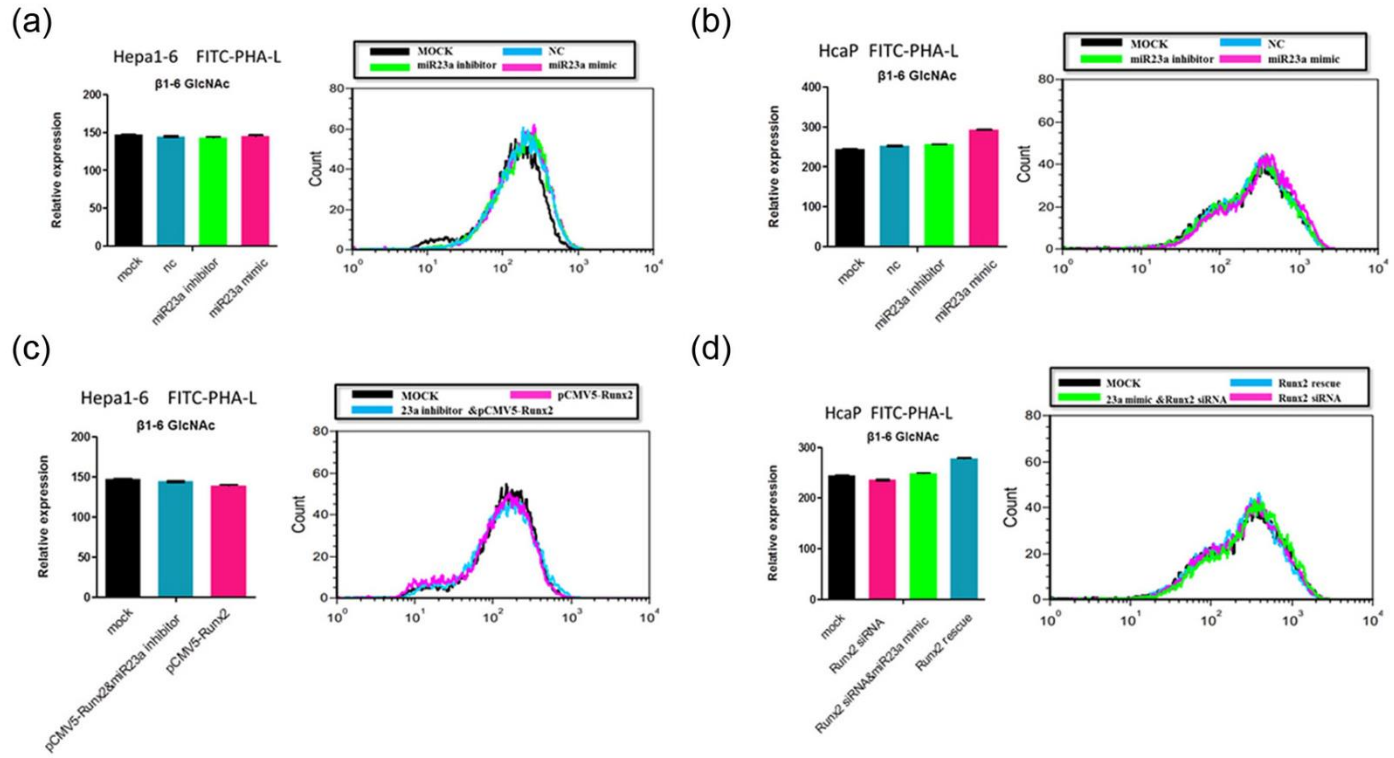

Supplementary Figure 2: Neither miR-23a nor Runx2 affects the levels of β-1,6 branching of N-glycans

(a) and (b) FCM analysis of the levels of β-1,6 branching of N-glycans recognized by FITC-PHA-L on the cell surface of Hepa1-6 (a) and Hca-P (b) cells transfected with miR-23a mimic or miR-23a inhibitor relative to CP transfection reagent only (mock). No significant difference was detected. (c) FCM analysis of the levels of β-1,6 branching of N-glycans recognized by FITC-PHA-L on the cell surface of Hepa1-6 cells transfected with 3.6 μg of pCMV-Runx2 plasmid or 150 nM miR-23a inhibitor and 3 μg pCMV-Runx2 plasmid relative to CP transfection reagent only (mock). (d) FCM analysis of the levels of β-1,6 branching of N-glycans recognized by FITC-PHA-L on the cell surface of Hca-P cells transfected with 100 nM Runx2 siRNA, 100 nM Runx2 siRNA and 3 μg pCMV-Runx2 plasmid (Runx2 rescued after 24 h), or 150 nM miR-23a mimic and 100 nM Runx2 siRNA relative to CP transfection agent only (mock). No significant difference was detected. Data are presented as the median with error bars.

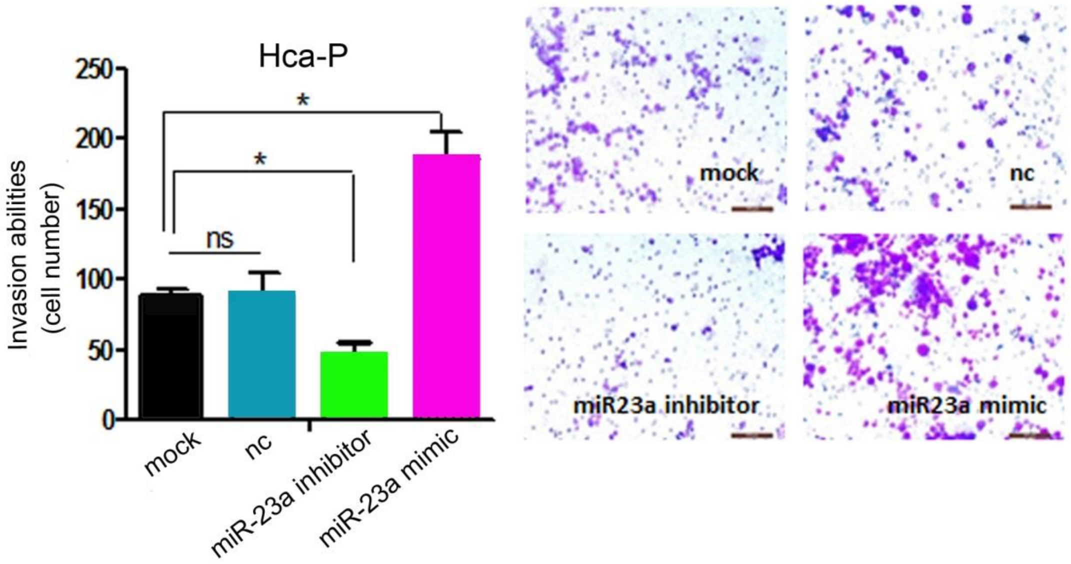

Supplementary Figure 3: miR-23a promotes Hca-P cell invasion in vitro Transwell migration assay of Hca-P cells transfected with CP transfection reagent (mock), scrambled miRNA (NC), miR-23a mimic or miR-23a inhibitor. Representative pictures of migrated Hca-P cells (right) and quantification of the number of invaded tumor cells (left). The fields of view were randomly selected under a microscope, and the micrograph scale bar represents 100 μm.

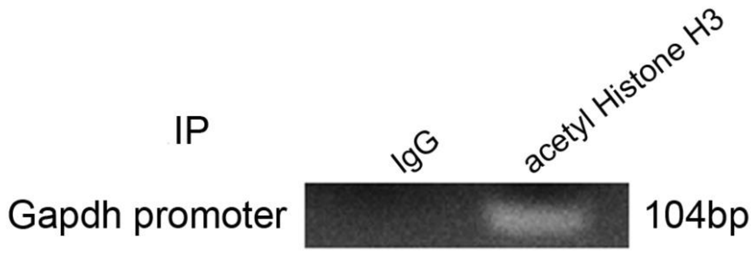

Supplementary Figure 4: ChIP assay positive control

ChIP assays were performed according to the EZ-Magna ChIP^{TM} protocol using chromatin from Hepa1-6 cells, and both anti-acetyl Histone H3 and normal rabbit IgG were used as the immunoprecipitating antibodies during an overnight incubation. Purified DNA was then analyzed by RT-PCR using primers specific for the Gapdh promoter.

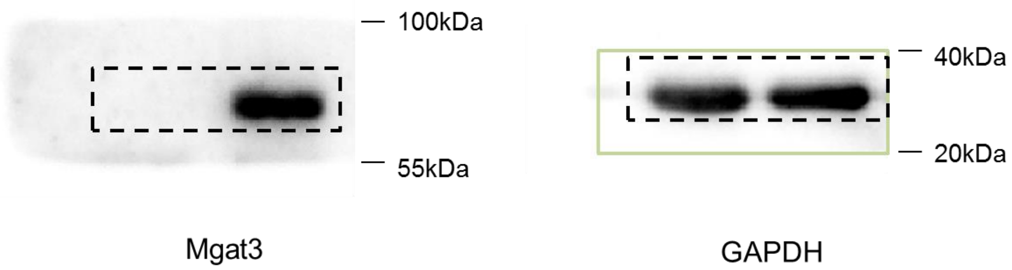

Supplementary Figure 5: The whole membrane staining for the western blot data in Figure 1c, the PVDF membrane was cut according to protein marker after western transfer. Dashed lines indicate how images have been cropped for main figures. Green lines indicate the location of membrane, when it was hard to separate the membrane edges from background.

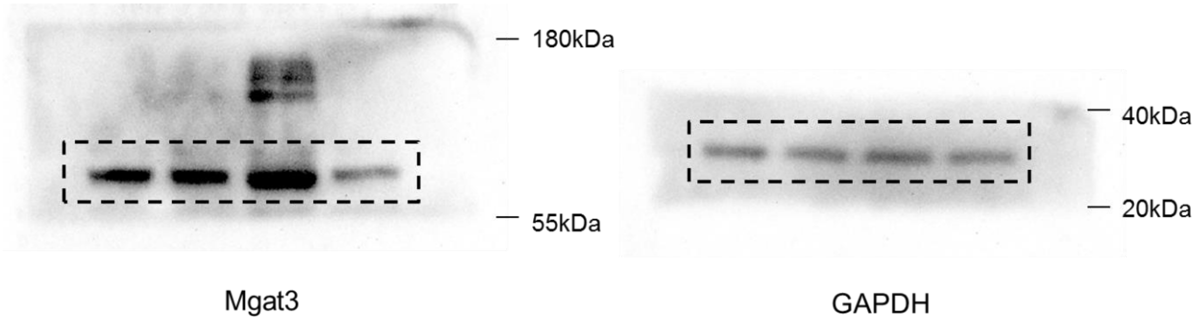

Supplementary Figure 6: The whole membrane staining for the western blot data in Figure 2c, the PVDF membrane was cut according to protein marker after western transfer. Dashed lines indicate how images have been cropped for main figures.

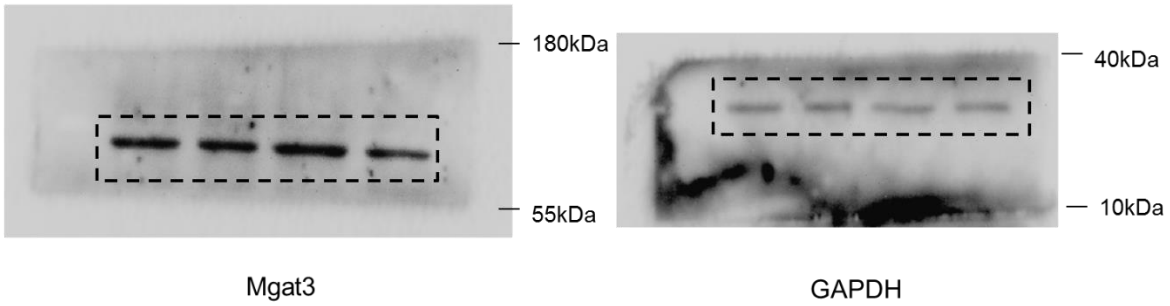

Supplementary Figure 7: The whole membrane staining for the western blot data in Figure 2d, the PVDF membrane was cut according to protein marker after western transfer. Dashed lines indicate how images have been cropped for main figures.

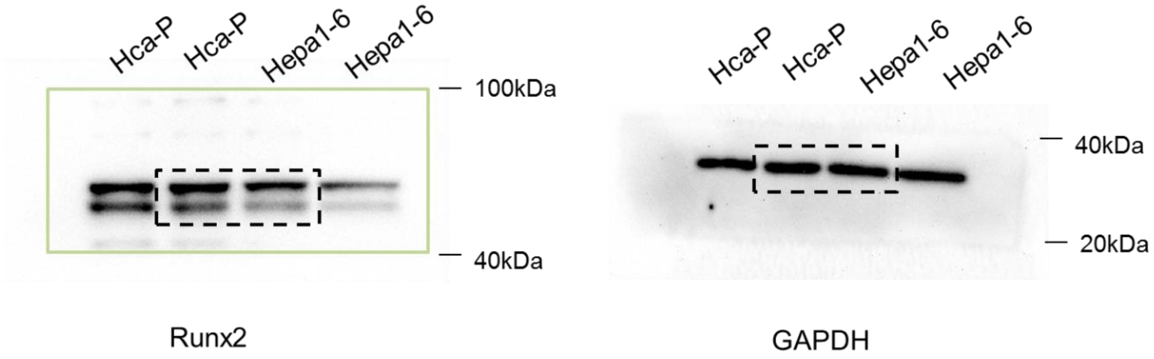

Supplementary Figure 8: The whole membrane staining for the western blot data in Figure 4a, the PVDF membrane was cut according to protein marker after western transfer. Dashed lines indicate how images have been cropped for main figures. The samples were loaded twice respectively. Green lines indicate the location of membrane, when it was hard to separate the membrane edges from background.

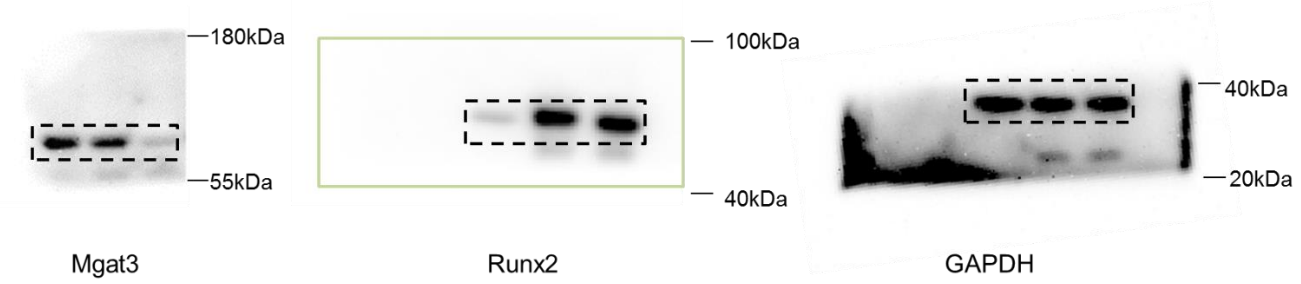

Supplementary Figure 9: The whole membrane staining for the western blot data in Figure 5a, the PVDF membranes were cut according to protein marker after western transfer. Dashed lines indicate how images have been cropped for main figures. Green lines indicate the location of membrane, when it was hard to separate the membrane edges from background.

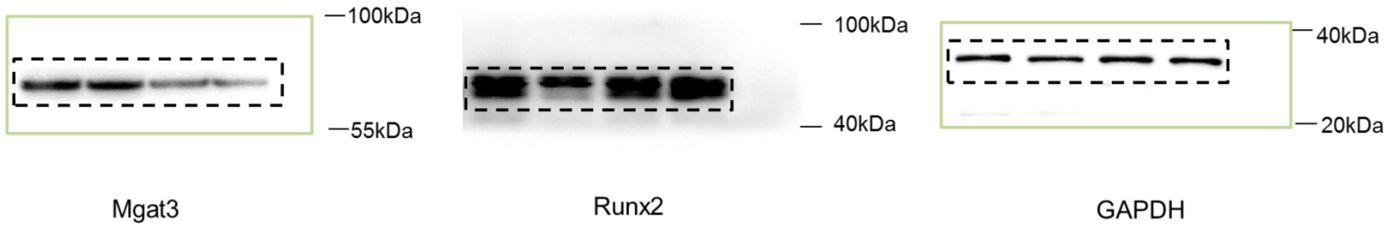

Supplementary Figure 10: The whole membrane staining for the western blot data in Figure 5b, the PVDF membranes were cut according to protein marker after western transfer. Dashed lines indicate how images have been cropped for main figures. Green lines indicate the location of membrane, when it was hard to separate the membrane edges from background.

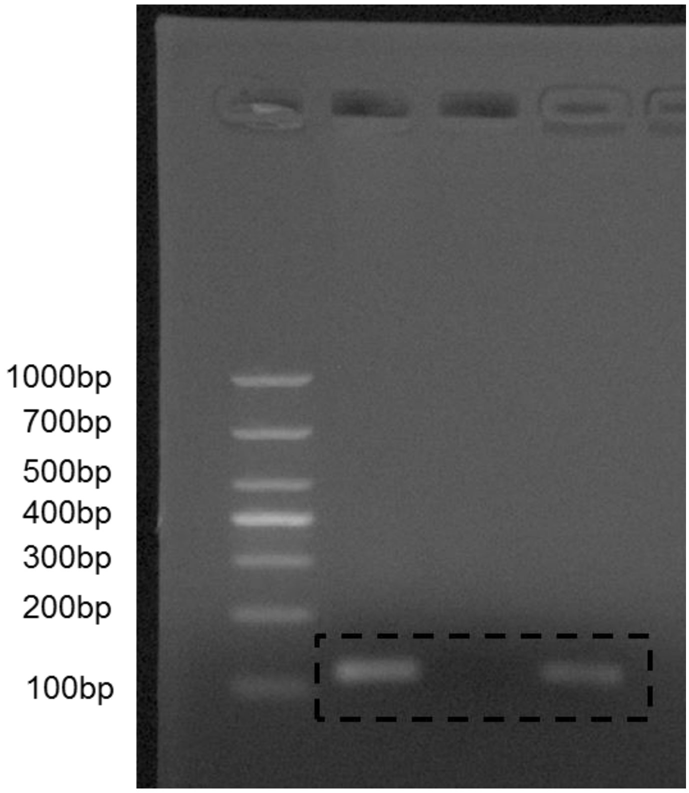

Supplementary Figure 11: Un-cut DNA electrophoretogram in Figure 4d. Dashed lines indicate how images have been cropped for main figures.
